# Genomic Characterization of Prevalent *mcr-1*, *mcr-4*, and *mcr-5 Escherichia coli* Within Swine Enteric Colibacillosis in Spain

**DOI:** 10.3389/fmicb.2019.02469

**Published:** 2019-11-01

**Authors:** Isidro García-Meniño, Dafne Díaz-Jiménez, Vanesa García, María de Toro, Saskia C. Flament-Simon, Jorge Blanco, Azucena Mora

**Affiliations:** ^1^Laboratorio de Referencia de Escherichia coli, Departamento de Microbiología y Parasitología, Facultad de Veterinaria, Universidad de Santiago de Compostela, Lugo, Spain; ^2^Plataforma de Genómica y Bioinformática, Centro de Investigación Biomédica de La Rioja, Logroño, Spain

**Keywords:** *Escherichia coli*, colistin, *mcr*, ESBL, fluoroquinolones, ST10, colibacillosis, swine

## Abstract

Antimicrobial agents are crucial for the treatment of many bacterial diseases in pigs, however, the massive use of critically important antibiotics such as colistin, fluoroquinolones and 3rd–4th-generation cephalosporins often selects for co-resistance. Based on a comprehensive characterization of 35 colistin-resistant *Escherichia coli* from swine enteric colibacillosis, belonging to prevalent Spanish lineages, the aims of the present study were to investigate the characteristics of *E. coli* clones successfully spread in swine and to assess the correlation of the *in vitro* results with *in silico* predictions from WGS data. The resistome analysis showed six different *mcr* variants: *mcr-1.1*; *mcr-1.10*; *mcr-4.1*; *mcr-4.2*; *mcr-4.5;* and *mcr-5.1*. Additionally, *bla*_CTX–M–__14_, *bla*_CTX–M–__32_ and *bla*_SHV–__12_ genes were present in seven genomes. PlasmidFinder revealed that *mcr-1.1* genes located mainly on IncHI2 and IncX4 types, and *mcr-4* on ColE10-like plasmids. Twenty-eight genomes showed a *gyrA* S83L substitution, and 12 of those 28 harbored double-serine mutations *gyrA* S83L and *parC* S80I, correlating with *in vitro* quinolone-resistances. Notably, 16 of the 35 *mcr-*bearing genomes showed mutations in the PmrA (S39I) and PmrB (V161G) proteins. The summative presence of mechanisms, associated with high-level of resistance to quinolones/fluoroquinolones and colistin, could be conferring adaptive advantages to prevalent pig *E. coli* lineages, such as the ST10-A (CH11-24), as presumed for ST131. SerotypeFinder allowed the H-antigen identification of *in vitro* non-mobile (HNM) isolates, revealing that 15 of the 21 HNM *E. coli* analyzed were H39. Since the H39 is associated with the most prevalent O antigens worldwide within swine colibacillosis, such as O108 and O157, it would be probably playing a role in porcine colibacillosis to be considered as a valuable subunit antigen in the formulation of a broadly protective Enterotoxigenic *E. coli* (ETEC) vaccine. Our data show common features with other European countries in relation to a prevalent clonal group (CC10), serotypes (O108:H39, O138:H10, O139:H1, O141:H4), high plasmid content within the isolates and *mcr* location, which would support global alternatives to the use of antibiotics in pigs. Here, we report for first time a rare finding so far, which is the co-occurrence of double colistin-resistance mechanisms in a significant number of *E. coli* isolates.

## Introduction

Multidrug-resistant Enterobacteriaceae, such as *Escherichia coli*, represent a threat to both human and veterinary health. *E. coli* has a great capacity to accumulate resistance genes, mostly through horizontal gene transfer. The major problematic mechanisms correspond to the acquisition of genes coding for extended-spectrum beta-lactamases (ESBL), carbapenemases, 16S rRNA methylases, plasmid-mediated quinolone resistance (PMQR) and *mcr* genes conferring resistance to polymyxins (Poirel et al., 2018).

Colistin has been widely used in Spain for the control of neonatal and post-weaning diarrhoea (PWD) in pigs caused by certain *E. coli* pathotypes: Enterotoxigenic *E. coli* (ETEC), defined by the presence of genes encoding enterotoxins (*eltA*, and/or *estA*, and/or *estB*); atypical Enteropathogenic *E. coli* (aEPEC), carriers of *eae* but negative for *bfpA* (aEPEC); Shiga toxin–producing *E. coli* (STEC), positive for *stx*_2__e_; STEC/ETEC, positive for both shiga toxin type 2e and enterotoxin-encoding genes (*stx*_2__e_ and *estB* and/or *estA*) (García-Meniño et al., 2018). PWD results in significant economic losses for the pig industry due to costs derived of treatment and handling, decreased weight gain, and mortality. These circumstances have promoted the use and abuse of antibiotics in intensive farming (Luppi, 2017; Rhouma et al., 2017). However, specific regulations have been set up in Europe due to the concern that colistin resistance could be transmitted from food-production animals to humans which makes necessary the investigation of sustainable alternatives to antimicrobials (EUROPEAN COMMISSION, 2018).

In Spain, the rates of antibiotic resistance in pig farming were recently analyzed in a collection of 499 *E. coli* isolates from 179 outbreaks of enteric colibacillosis occurred during a period of 10 years (2006–2016) (García et al., 2018; García-Meniño et al., 2018). The results revealed a prevalence of colistin-resistant *E. coli* implicated in PWD in Spanish farms as high as 76.9% within 186 ETEC, STEC and STEC/ETEC isolates. Besides, PCR and sequencing identified the presence of *mcr-4* in 102 isolates, *mcr-1* in 37 isolates and *mcr-5* in five isolates. Interestingly, almost all *mcr-4* isolates belonged to the clonal group ST10-A (CH11-24) (García et al., 2018), which was shown to be highly present (more than 50%) within the *mcr-1* diarrheagenic isolates of a second study (García-Meniño et al., 2018). Both studies reinforced other countries’ findings that the pig industry is an important reservoir of colistin-resistant *E. coli*, as well as being carriers of other additional risk genes such as *bla*_ESBL_ genes (García et al., 2018; García-Meniño et al., 2018; Magistrali et al., 2018; Manageiro et al., 2019). Based on reported evidences (Beyrouthy et al., 2017; Gilrane et al., 2017), there is great concern about the *in vivo* acquisition of *mcr-* and *bla*_ESBL_-bearing plasmids by human *E. coli* isolates following treatment with colistin, or via animal transmission through direct contact or via food chain. Particular attention is given to those named as high-risk clones of (ESBL)-producing bacteria, worldwide spread within humans and animals, including *Escherichia coli* sequence types ST10, ST131, ST405, and ST648 (Mathers et al., 2015; Sellera and Lincopan, 2019).

The aims of this study were (i) the characterization of resistances and plasmid profiles of successfully spread *mcr-1*, *mcr-4*, and *mcr-5 E. coli* in Spanish pig farming; (ii) the assessment of WGS-based approaches for the characterization of pathogenic *E. coli*, through the correlation of the *in vitro* results with *in silico* predictions using the bioinformatics tools of the Center for Genomic Epidemiology (CGE).

## Materials and Methods

### *E. coli* Collection

Thirty-five swine *E. coli*, positive by PCR for *mcr-*genes, were fully sequenced. Specifically, the 35 *E. coli* were selected from 499 diarrheagenic isolates of different geographic areas of Spain (2006–2016) (García et al., 2018; García-Meniño et al., 2018), taking into account the results of prevalence and significant association observed between pathotypes, presence of *mcr* and certain serogroups. In brief, the serogroups O108, O138, O141, O149, O157 were found significantly associated with ETEC; serogroups O26, O49, O80, O111 with aEPEC; serogroups O138 and O141 with STEC/ETEC; serogroup O139 with STEC; and serogroups O2, O15, O26, O45, O111, O138, O141, O157 with *mcr*-positive isolates (García-Meniño et al., 2018). Therefore, the collection analyzed here included 27 ETEC isolates (of serogroups O7, O8, O15, O45, O108, O138, O141, O149, O157, ONT); four STEC (O2, O139); three STEC/ETEC (O138 and O141) and one aEPEC (O111). The 35 representative isolates were carriers of the three *mcr*-types (*mcr-1*, *mcr-4*, and *mcr-5*) detected so far in our *E. coli* collection of porcine origin. Conventional pheno- and geno-typing was performed to complete classical characterization of serotypes, phylogroups, pathotypes and resistance profiles.

### Conventional Typing

The H antigen was established for motile isolates by serotyping using H1 to H56 antisera, while non-motile isolates (HNM) were analyzed by PCR to determine their flagellar genes as described elsewhere (García-Meniño et al., 2018). The phylogroup was assigned by means of the quadruplex PCR of Clermont et al. (2013). Antimicrobial susceptibility was determined by minimal inhibitory concentrations (MICs) using the MicroScan WalkAway^®^-automated system (Siemens Healthcare Diagnostics, Berkeley, CA, United States) according to the manufacturer’s instructions for: amikacin, ampicillin-sulbactam, aztreonam, cefepime, ceftazidime, ciprofloxacin, colistin, fosfomycin, gentamicin, imipenem, levofloxacin, meropenem, minocycline, nitrofurantoin, piperacillin-tazobactam, ticarcillin, tigecycline, and tobramycin. Additionally, resistance to ampicillin, amoxicillin/clavulanate, cefazolin, cefotaxime, cefoxitin, cefuroxime, chloramphenicol, doxycycline, nalidixic acid and trimethoprim-sulfamethoxazole was determined by disk (Becton Dickinson, Sparks, MD, United States) diffusion assays. All results were interpreted according to the CLSI break points (Clinical and Laboratory Standards Institute, 2019). Genetic identification of the ESBLs was performed by PCR using the TEM, SHV, CTX-M-1 and CTX-M-9 group-specific primers followed by amplicon sequencing (García-Meniño et al., 2018).

### Whole Genome Sequencing (WGS) and Sequence Analysis

The libraries for sequencing were prepared following the instructions provided by the TruSeq Illumina PCR-Free protocol. Mechanical DNA fragmentation was performed with Covaris E220, and the final quality of the libraries assessed with Fragment Analyzer (Std. Sens. NGS Fragment Analysis kit 1-6000 bp). Lastly, the libraries were sequenced in an Illumina HiSeq1500, obtaining 100–150 bp paired-end reads which were trimmed (Trim Galore 0.5.0) and filtered according to quality criteria (FastQC 0.11.7). The reconstruction of the genomes and plasmids in the genomes was carried out using the methodology PLAsmid Constellation NETwork (PLACNETw)^1^ (Lanza et al., 2014). The assembled contigs, with genomic size ranging between 4.9 and 5.9 Mbp (mean size 5.5 Mbp), were analyzed using the bioinformatics tools of the Center for Genomic Epidemiology (CGE)^2^ for the presence of antibiotic resistance (ResFinder V2.1.), virulence genes (VirulenceFinder v1.5.), plasmid replicon types (PlasmidFinder 1.3./PMLST 1.4.), and identification of clonotypes (CHTyper 1.0), sequence types (MLST 2.0) and serotypes (SerotypeFinder 2.0). All the CGE predictions were called applying a select threshold for identification and a minimum length of 95 and 80%, respectively. Phylogroups were predicted using the ClermonTyping tool at the iame-research center web^3^. The *mcr* gene location was determined using PlasmidFinder/ResFinder prediction, together with PLACNETw references, and automatic annotation with Prokka v1.13 (Seemann, 2014).

## Results and Discussion

The phenotypic and genotypic traits of the 35 *mcr*-positive *E. coli* of swine origin, as well as their resistome and mobilome are summarized in Table 1. ResFinder confirmed that all genomes were *mcr* carriers. Likewise, VirulenceFinder predicted the acquired virulence genes encoding for the enterotoxins (*sta1*, *stb*, *itcA*), for fimbriae (*fedF*, *k88*), verotoxin (*stx2*) and intimin (*eae*), correlating in all cases with the pathotype assignation previously determined by PCR (García et al., 2018; García-Meniño et al., 2018).

**TABLE 1:** Features of the 35 colistin-resistant *E. coli* genomes of swine origin based on *in silico* characterization (light columns) and on conventional typing (gray columns).

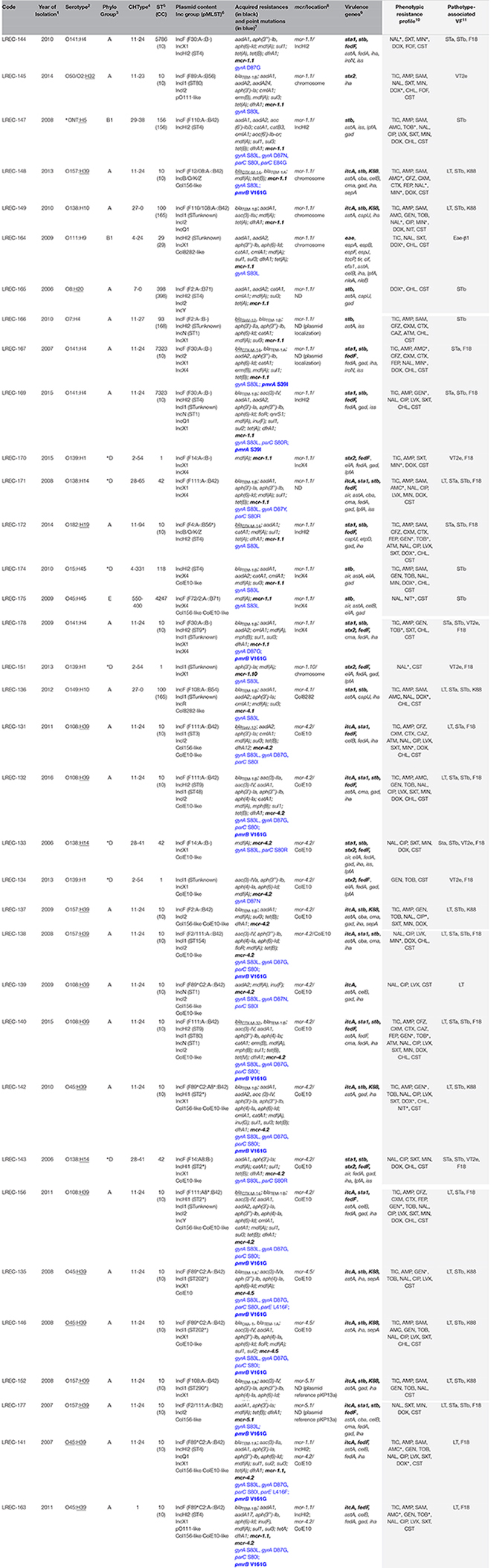									

### Serotype Identification

In most studies, there is lack of information on *E. coli* serotypes since serotyping is performed by very few laboratories worldwide, hindering epidemiological comparisons. Here, we not only proved that there is a very good correlation between serotyping and SerotypeFinder predictions, but also the advantage of *in silico* H-antigen identification for those non-mobile (HNM) isolates. It is of note that 15 of the 21 HNM isolates were predicted as H39 (Table 1), namely O108:H39, O157:H39 and O45:H39 (five genomes, each). Given that the H39 is associated with the most prevalent O antigens within swine colibacillosis, such as O108 and O157, as well as ONT (García-Meniño et al., 2018), it would be probably playing a role in porcine colibacillosis to be considered as a valuable subunit antigen in the formulation of a broadly protective ETEC vaccine (Roy et al., 2009). The remaining six HNM isolates showed different O:H combinations: O138:H14, ONT:H5, O8:H20, O50/O2:H32, and O182:H19. SerotypeFinder also allowed the O45-antigen determination of two non-typeable (ONT) isolates (LREC-141 and LREC-146) and O182 of LREC-172; while LREC-147, belonging to O157 serogroup (Table 1), was predicted as ONT, probably due to the limitation of the assembly based on Illumina short reads (100–150 bp paired-end reads here) (Wick et al., 2017).

### Phylogroups, Sequence Types and Clonotypes

The phylogroups established for the 35 genomes were the common ones reported for porcine *E. coli* isolates (A, B1, D-E) (Shepard et al., 2012; Bosak et al., 2019). However, we found discrepancies in the assignation obtained with the quadruplex PCR of Clermont et al. (2013) in comparison with that predicted by ClermonTyping for seven isolates: phylogroup E by PCR, while phylogroup D *in silico* (Table 1).

MLST and CHTyper tools determined 12 different STs, but mostly belonging to CC10 (21 genomes) and clonotype CH11-24 (18 genomes) (Table 1). The predominance of CC10, and specifically ST10, is in accordance with published data on *E. coli* isolates of swine origin, independently of the pathogenicity or antibiotic-resistance/susceptibility status (Shepard et al., 2012; Kidsley et al., 2018; Magistrali et al., 2018).

### Resistome, Plasmidome and Phenotypic Expression of Resistances

The resistome analysis revealed that 34 of the 35 genomes encoded mechanisms of antibiotic resistance for ≥three different antimicrobial categories (Table 1). Seven *E. coli* were carriers of *bla*_ESBL_, namely *bla*_CTX–M–__14_ (four genomes), *bla*_CTX–M–__32_ (one) and *bla*_SHV–__12_ (two). Besides, six different *mcr* variants were identified within the 35 *E. coli*: *mcr-1.1* (in 18 genomes, including two *mcr-4.2* carriers); *mcr-1.10* (one); *mcr-4.1* (one); *mcr-4.2* (13 genomes, including the two *mcr-1.1* carriers); *mcr-4.5* (two) and *mcr-5.1* (two).

PlasmidFinder revealed a high plasmid diversity based on the identified replicons, with two to seven different plasmid types per genome (Table 1). Within this heterogeneity, *mcr-1.1* genes were found mainly on plasmids of the IncHI2 and IncX4 types (six and four of the 12 *mcr-1.1* plasmid-located genes, respectively); however, *mcr-1.1* was also found integrated in the chromosome of LREC-145, LREC-148, LREC-149 and LREC-164 genomes. The *mcr-1.10* gene of LREC-151 was located on the chromosome, while *mcr-4* and *mcr-5* variants were on Col8282-like (*mcr-4.1*), ColE10-like (for all 13 *mcr-4.2* and two *mcr-4.5* carriers) and pKP13a-like (*mcr-5.1*) plasmids. Furthermore, we found that there was no *mcr* plasmid co-occurrence in LREC-141 and LREC-163, but rather the *mcr-1.1* and *mcr-4.2* genes were located in independent plasmids (IncHI2 and ColE10-like types, respectively). The *mcr* location remained undetermined for four isolates.

Since the *mcr-1* plasmid gene was first described (Liu et al., 2016), it has been identified in members of the Enterobacteriaceae family encoded in different plasmid types, including IncI2, IncX4, IncHI1, IncHI2, IncFI, IncFII, IncP, IncK (Sun et al., 2018). Different authors corroborate that large conjugative plasmids of types IncHI2, IncX4 and IncI2 would be the maximum responsible for the dissemination of the *mcr-1* gene among *E. coli* isolates from different sources and geographical locations (Doumith et al., 2016; Li et al., 2017; Manageiro et al., 2019). To date, other *mcr* genes (2–9) have been described (Carroll et al., 2019); among them, the *mcr-4* and *mcr-5* genes appear mostly encoded in small and non-conjugative ColE-like type plasmids (Sun et al., 2018). Here we found similar results, since *mcr-1.1* genes were located mainly on IncHI2 and IncX4 types, and *mcr-4* on ColE10-like plasmids. It is of note that the *mcr-5.1* gene, predicted in LREC-152 and LREC-177, was linked to a Kp13-like plasmid (CP003996.1), location previously described by Hammerl et al. (2018) for one *mcr-5* isolate recovered from a fecal pig sample at farm. Chromosomally-encoded *mcr-1* location remains rare, however, it was described soon after the discovery of this plasmid-borne gene (Falgenhauer et al., 2016; Veldman et al., 2016). Here, we determined chromosomal location in five genomes by means of PLACNETw, and according to the predictive annotation of the *mcr*-contigs, the only common element flanking the *mcr-1* was a putative ORF, *pap2*, which is part of the Tn*6360* and encodes a Pap2 superfamily protein. Thus, Pap2 was detected in LREC-145, LREC-148, LREC-149, and LREC-164, while the IS*ApI1* element typically associated with the initial mobilization of *mcr-1*, was missing within the five contigs (Snesrud et al., 2018).

Overall, our findings are in accordance with those reported by Magistrali et al. (2018) on 13 *mcr-*positive *E. coli* isolated from swine colibacillosis in Belgium, Italy and Spain. Both studies show common features in relation to a prevalent clonal group (CC10), serotypes (O108:H39, O138:H10, O139:H1, O141:H4), and *mcr*-plasmid types. The confirmation of these similarities are of interest for the global design of alternatives to antibiotics that would curb the dissemination of specific clones in the pig farming.

The *in vitro* analysis of resistances showed that 30 of the 35 *E. coli* were multidrug-resistant (MDR) according to Magiorakos et al. (2012) definition (Table 1). Phenotypic results corresponded broadly to those predicted by ResFinder (Supplementary Table S1) as detailed below.

The quinolones/fluoroquinolones (FQ), together with polymyxins and 3rd–4th-generation cephalosporins, all are included in Category B of restricted antimicrobials in the EMA categorization, considering that the risk to public health resulting from its veterinary use needs to be mitigated by specific restriction (EMA/CVMP/CHMP, 2019). Two major mechanisms are implicated in the resistance to FQ, namely, mutations in the genes for DNA gyrase and topoisomerase IV, and decreased intracellular drug accumulation. In addition, plasmid-mediated quinolone resistances also play a role but usually conferring low-level FQ resistance (van Duijkeren et al., 2018). Phenotypically, 17 of the 35 isolates showed resistance to both nalidixic acid and ciprofloxacin, and other eight resistance to nalidixic acid only (Supplementary Table S1). In the majority of cases, phenotypic results correlated with those predicted by ResFinder. Particularly, 28 of the 35 genomes carried the *gyrA* S83L substitution, with 12 of those 28 showing double-serine mutations (*gyrA* S83L and *parC* S80I). An additional substitution (*gyrA* D87N) was detected in two of the 12 *gyrA* S83L/*parC* S80I genomes. Thus, nalidixic acid resistance *in vitro* corresponded to one single substitution (*gyrA* S83L), and FQ resistance to double or triple substitutions (*gyrA* S83L/*parC* S80I/*gyrA* D87N). Plasmid-mediated quinolone resistant genes *acc(6′)-Ib-cr* and *qnrS1* were also present together with chromosomal mutations in LREC-147 and LREC-169, respectively. Double-serine mutations in specific positions of the *gyrA* and *parC* genes have been reported as a dominant feature of MDR lineages within *E. coli*, *S. aureus* and *K. pneumoniae*, with favorable fitness balance linked to high levels of resistance to FQ (Fuzi et al., 2017). This finding, in 12 out of the 28 *in silico* predicted FQ-resistant could be conferring adaptive advantages to certain widely spread pig pathogenic clonal groups of *E. coli*, such as the ST10-A (CH11-24) (García et al., 2018). This hypothesis is presently assumed for ST131 and other risk clones linked to high FQ-resistance (Johnson et al., 2015; Fuzi et al., 2017).

On the other hand, colistin has been widely used for the control of enteric diseases, mainly in swine and poultry (Rhouma et al., 2016; Hammerl et al., 2018). Several mechanisms of resistance due to chromosomal mutations or acquired resistance genes have been described so far (Olaitan et al., 2014; Poirel et al., 2018). The 35 colistin-resistant *E. coli* of this study showed MIC values > 4 mg/L. As detailed above, ResFinder confirmed that all the analyzed genomes were *mcr*-carriers. In addition to the plasmid mechanism (*mcr*) of resistance, polymyxin resistance in *E. coli* can be due to genes encoding LPS-modifying enzymes, particularly to mutations in the two-component systems PmrAB and PhoPQ, or in the MgrB regulator. Quesada et al. (2015) detected two colistin-resistant *E. coli* recovered in 2011 and 2013 from the stools of two pigs, which showed mutations in PmrB V161G and PmrA S39I, reporting the finding as a rare event. Subsequently, Delannoy et al. (2017) analyzed 90 strains of *E. coli* isolated from diseased pigs: 81 were phenotypically resistant to colistin and 72 *mcr-1* carriers (including two colistin-susceptible). Although different mutations were found in the amino acid sequences of the MgrB, PhoP, PhoQ, and PmrB proteins of eight isolates, only two of them were *mcr-1* positive (but colistin-susceptible). Surprisingly, we found here the double mechanism of colistin resistance in 16 *E. coli*, harboring *mcr*-genes together with one amino acid substitution: PmrB V161G (14 genomes) or PmrA S39I (two genomes). In a recent study on Parisian inpatient fecal *E. coli* (Bourrel et al., 2019), the authors found 12.5% of colistin-resistant *E. coli* carriers among 1,217 patients; however, *mcr-1* gene was identified in only seven of 153 isolates, while 72.6% harbored mutations in the PmrA and PmrB proteins. According to the authors, their findings indicate two evolutionary paths leading to colistin resistance in human fecal *E. coli*, one corresponding to a minority of plasmid-encoded *mcr-1* isolates of animal origin, and a second corresponding to a vast majority of human isolates exhibiting chromosomally encoded mechanisms (Bourrel et al., 2019). Thus, and given the limited data regarding the co-occurrence of double resistance mechanism, it is of note that 16 of the 35 *mcr-*bearing genomes of our study showed mutations in the PmrA and PmrB proteins. Furthermore, two *E. coli* (LREC-141 and LREC-163) shown to be carriers of two different *mcr-*bearing plasmids together with PmrB V161G mutation. An explanation for this rare finding is that these isolates would be reflecting a cumulative evolution to antibiotic pressure and, as a consequence, enhancing the transmission (vertical and horizontal) of colistin resistance. In any case, further investigation is needed to evaluate the implication of chromosomal mutations and *mcr* co-occurrence regarding colistin resistance phenotype.

In this study, 22 out of the 25 isolates showing phenotypic resistance to beta-lactams (Supplementary Table S1), were positive in the analysis *in silico* for the presence of *bla*_TEM–__1_ genes, alone (14 genomes), or in combination with other *bla* genes (*bla*_CTX–M–__14_, *bla*_SHV–__12_, *bla*_CTX–M–__32_ and *bla*_OXA–__1_); additionally, two genomes showed the presence of *bla*_CTX–M–__14_ and *bla*_SHV–__12_, respectively. With the exception of LREC-147, LREC-164, and LREC-170, which were phenotypically resistant to narrow-spectrum beta-lactamases but negative for the presence of genes, a good correlation was observed between genes predicted and resistance shown *in vitro*. It is of note that *bla*_TEM–__135_, determined in LREC-156 by conventional typing, was not identified *in silico*. Beta-lactams are the most widely used family in current clinical practice. Numerous genes in *E. coli* confer resistance to this group, being some of them, such as *bla*_TEM–__1_ widespread in *E. coli* from animals coding for narrow-spectrum beta-lactamases that can inactivate penicillins and aminopenicillins. However, genes encoding ESBLs/AmpCs have increasingly emerged in *E. coli* from humans and animals, including food-producing animals (Cortes et al., 2010).

Thirty out of the 35 genomes showed high frequency of resistance genes to aminoglycosides, specifically encoding AAC(3)-II/IV and AAC(6)-Ib, which are the most frequently encountered acetyltransferases among *E. coli* of human and animal origins. The subclass AAC(3)-II, which is characterized by resistance to gentamicin, netilmicin, tobramycin, sisomicin, 2′-*N*-ethylnetilmicin, 6′-*N*-ethylnetilmicin and dibekacin (Shaw et al., 1993), and AAC(6′) enzymes that specify resistance to several aminoglycosides and differ in their activity against amikacin and gentamicin C1 (Ramirez and Tolmasky, 2010) seemed to correlate with the phenotypic detection of resistance to gentamicin and/or tobramycin (12 of the 17 resistant isolates) (Supplementary Table S1). We also detected high prevalence of genes encoding nucleotidyltransferases (*aadA*), which specify resistance to spectinomycin and streptomycin, alone or together with phosphotransferases (APHs) (Ramirez and Tolmasky, 2010), but they were not tested in the phenotypic antimicrobial susceptibility tests.

It is noteworthy that the 35 genomes of our study were carriers of *mdf(A)*. Edgar and Bibi (1997) described that cells expressing MdfA from a multicopy plasmid are substantially more resistant to a diverse group of cationic or zwitterionic lipophilic compounds. Besides, the authors found that MdfA also confers resistance to chemically unrelated, clinically important antibiotics such as chloramphenicol, erythromycin, and certain aminoglycosides and fluoroquinolones. This capability could correlate with the *in vitro* resistance observed for some isolates to tetracyclines and aminoglycosides, in absence of other specific genes. In our collection, of the 24 isolates showing phenotypic resistance to minocycline and, or doxycycline (Supplementary Table S1), 20 showed carriage of *tet* genes: 12 *tet(B)*, six *tet(A)*, one *tet(A)* + *tet(B)* and one *tet(B)* + *tet(M)*. However, two *tet(A)* isolates were susceptible to those antibiotics (LREC-163, LREC-169). Additionally, *tet* genes were not detected *in silico* in four phenotypically resistant isolates. In general, *tet(A)* and *tet(B)* are the most prevalent tetracycline resistance genes in *E. coli* of animal origin, and specifically in isolates from pigs (Tang et al., 2011; Holzel et al., 2012; Jurado-Rabadan et al., 2014).

Although the use of chloramphenicol was banned in the European Union in food-producing animals in 1994, fluorinated derivative florfenicol is allowed for the treatment of bacterial infection in these animals (Schwarz et al., 2004; OIE, 2019). In the present study, all 19 chloramphenicol-resistant isolates (Supplementary Table S1) correlated with the presence of genes *catA1* (12 genomes), *catB3* (one genomes), *cmlA* (ten genomes) or *floR* (three genomes) detected *in silico*. Travis et al. (2006) showed that chloramphenicol resistant genes are frequently linked to other antibioresistance genes. Thus, through transformation experiments conducted with *E. coli* from pigs demonstrated that *aadA* and *sul1* were located with *catA1* on a large ETEC plasmid, and plasmids carrying *cmlA* also carried *sul3* and *aadA*. According to the authors, this linkage might partly explain the long-term persistence of chloramphenicol resistance in ETEC despite its withdrawal years ago. In our study, ResFinder also showed an association of genes *cmlA, sul3* and *aadA* present in the same contig (7 of the 10 genomes positive for *cmlA*), and *cmlA/aadA* in all cases. Additionally, *aadA* and *sul1* were located with *floR* in LREC-146.

In *E. coli* from food-producing animals, sulfonamide resistance is mediated by *sul* genes (*sul1, sul2, sul3*), widely disseminated, and frequently found together with other antimicrobial resistance genes, while *dfr* genes confer trimethoprim resistance in *E. coli* and other gram-negative bacteria (van Duijkeren et al., 2018). Within our collection, 20 of the 35 isolates were *in vitro* resistant to trimethoprim/sulfamethoxazole (Supplementary Table S1), and most of them correlated with the presence of *sul* + *dfrA* genes in their genomes, with the exception of LREC-133 and LREC-170 (negative for the *in silico* detection of *sul*, *dfrA* genes) and LREC-146 (in which only *sul1* and *sul2* genes were predicted). Besides, ResFinder showed that *sul1* (present in 16 genomes), *sul3* (14 genomes) and *sul2* (three genomes) were located together with *dfrA*, and other resistance genes, as mentioned previously.

The fosfomycin resistance showed *in vitro* by two isolates of the study collection, was not predicted for LREC-144 and LREC-145 (Supplementary Table S1) by ResFinder, which analyzes the presence of *fos* genes encoding for fosfomycin-modifying enzymes. The use of this antibiotic has been limited to the treatment of infections by Gram-positive and negative pathogens, included *E. coli*, mainly in pig and poultry farming (Poirel et al., 2018). However, phosphonic acid derivates such as fosfomycin, have been recently categorized by the EMA (EMA/CVMP/CHMP, 2019) as Category A (antimicrobial classes not currently authorized in veterinary medicine in EU).

## Conclusion

Swine colibacillosis control has been traditionally managed through the extensive use of antibiotics. Our results are a reflection of the situation within the industrial pig farming, where global hygiene procedures and vaccinations are essential for improvement in antimicrobial stewardship. The summative presence of antibioresistances could be conferring adaptive advantages to prevalent pig *E. coli* lineages, such as the ST10-A (CH11-24). Based on the different replicons identified by PlasmidFinder (up to seven), it is of note the high plasmid diversity found within these isolates; further research is needed to know mechanisms of maintenance and advantages conferred to them.

Here, we report for first time a rare finding so far, which is the co-occurrence of double colistin-resistance mechanisms (*mcr*-genes and chromosomal mutations in the PmrA and PmrB proteins) in a significant number of *E. coli* isolates. This fact could be increasing the risk of colistin resistance-acquisition by means of food transmission. Globally, we found a very good correlation between resistances determined *in vitro* and genes predicted using CGE tools, and the same observation applies to the *E. coli* pathotype determination.

## Data Availability Statement

The nucleotide sequence of the 35 LREC genomes have been deposited in the NCBI sequence databases with accession codes SAMN11523829 to SAMN11523863. These sequences are part of BioProject ID PRJNA540146.

## Author Contributions

IG-M, DD-J, and SF-S undertook the laboratory work. AM and JB conceived and designed the study. IG-M, DD-J, VG, MT, and AM performed the data analysis. IG-M, DD-J, VG, MT, JB, and AM drafted the manuscript. All authors provided critical input and approved the final version.

## Conflict of Interest

The authors declare that the research was conducted in the absence of any commercial or financial relationships that could be construed as a potential conflict of interest.

## Footnotes

1 https://castillo.dicom.unican.es/upload/

2 https://cge.cbs.dtu.dk/services/

3 http://clermontyping.iame-research.center/
