# Supplementary material for: Genomic Characterization of Prevalent mcr-1, mcr-4, and mcr-5 Escherichia coli Within Swine Enteric Colibacillosis in Spain
Source: Front Microbiol. 2019 Nov 1;10:2469. doi: 10.3389/fmicb.2019.02469 (PMC6838222; doi:10.3389/fmicb.2019.02469)
Supplement: Supplementary file 1 [file Table_1.DOCX]

Supplementary Material

**Genomic characterization of prevalent *mcr-1*, *mcr-4* and *mcr-5* *Escherichia coli* within swine enteric colibacillosis in Spain**

**Isidro García-Meniño^1,#^, Dafne Díaz-Jiménez^1,#^, Vanesa García^1,3^, María de Toro^2^, Saskia C. Flament-Simon^1^, Jorge Blanco^1^, Azucena Mora^1,*^**

*** Correspondence:** [azucena.mora@usc.es](mailto:azucena.mora@usc.es)

**Table S1.** ResFinder results for the 35 colistin-resistant *E. coli* compared with phenotypic resistance data

| **Code** | **Beta-lactam** | **Aminoglycosides** | **Macrolides** | **Tetracycline** | **Phenicols** | **Sulphonamides;**  **trimethoprim** | **FQ** | **Colistin** | **Others** |
| --- | --- | --- | --- | --- | --- | --- | --- | --- | --- |
|  | ResFinder/  phenotype^1^ | ResFinder/  phenotype^1^ | ResFinder/  phenotype^1,2^ | ResFinder/  phenotype^1^ | ResFinder/  phenotype^1^ | ResFinder/  phenotype^1^ | ResFinder/  phenotype^1^ | ResFinder/  phenotype^1^ | ResFinder/  phenotype^1^ |
| LREC-144 | - / - | *aadA1, aph(3'')-Ib, aph(6)-Id*  / - | *mdf(A)* | *tet(A), tet(B)*  */* MIN*, DOX | - / - | *sul1; dfrA1*  */* SXT | *gyrA D87G*  / NAL* | *mcr-1.1*  */* CST | -  / FOF |
| LREC-145 | *bla_TEM-1B_*  / TIC, AMP, SAM | *aadA1, aadA2, aadA24, aph(3')-Ia*  / - | *erm(B), mdf(A)* | *tet(A)*  */* MIN*, DOX | *cmlA1*  / CHL | *sul1; dfrA1*  */* SXT | *gyrA S83L*  / NAL | *mcr-1.1*  */* CST | -  / FOF |
| LREC-147 | -  / TIC, AMP, SAM, AMC | *aadA1, aadA2, acc(6')-Ib3*  / TOB* | *mdf(A)* | *tet(B)*  / MIN, DOX | *catA1, catB3, cmlA1*  / CHL | *sul1, sul3; dfrA1*  / SXT | *acc(6')-Ib-cr;*  *gyrA S83L, gyrA D87N, parC S80I, parC E84G*  / NAL, CIP, LVX | *mcr-1.1*  */* CST | - / - |
| LREC-148 | *bla_CTX-M-14_, bla_TEM-1A_*  / TIC, AMP, SAM, AMC*, CFZ, CXM, CTX, FEP | - / - | *mdf(A)* | *tet(B)*  */* MIN*, DOX | - / - | - / - | *gyrA S83L*  / NAL* | *mcr-1.1;*  *pmrB V161G*  */* CST | - / - |
| LREC-149 | *bla_TEM-1B_*  / TIC, AMP, SAM, AMC | *aadA1, aac(3)-IIa*  / GEN, TOB | *mdf(A)* | *tet(A)*  */* MIN*, DOX | - / - | *dfrA1*  */* - | *-*  / NAL*, CIP | *mcr-1.1*  */* CST | NIT |
| LREC-164 | -  / TIC | *aadA1, aadA2, aph(3'')-Ib, aph(6)-Id*  / - | *mdf(A)* | *tet(A)*  */* DOX* | *catA1, cmlA1*  / CHL | *sul1, sul3; dfrA1*  / SXT | *gyrA S83L*  / NAL | *mcr-1.1*  */* CST | - / - |
| LREC-165 | - / - | *aadA1, aadA2*  / - | *mdf(A)* | *tet(A)*  */* DOX* | *catA1, cmlA1*  / CHL | *sul3*  / - | - / - | *mcr-1.1*  */* CST | - / - |
| LREC-166 | *bla_SHV-12_, bla_TEM-1B_*  / TIC, AMP, SAM, CFZ, CXM, CTX, CAZ, ATM | *aph(3')-IIa, aph(3'')-Ib, aph(6)-Id*  / - | *mdf(A)* | - / - | *catA1*  / CHL | *sul3*  / - | - / - | *mcr-1.1*  */* CST | - / - |
| LREC-167 | *bla_CTX-M-14_, bla_TEM-1 A_*  / TIC, AMP, AMC*, CFZ,  CXM, CTX, FEP | *aadA2, aph(3'')-Ib, aph(6)-Id*  / - | *erm(B), mdf(A)* | *tet(B)*  / MIN*, DOX | - / - | *sul1*  / - | *gyrA S83L*  / NAL | *mcr-1.1;*  *pmrA S39I*  / CST | - / - |
| LREC-169 | *bla_TEM-1B_*  / TIC, AMP | *aac(3)-IV, aadA1, aadA2, aph(3')-Ia, aph(3'')-Ib, aph(6)-Id*  / GEN* | *mdf(A), inu(F)* | *tet(A)*  */ -* | *floR*  / CHL | *sul1, sul2; dfrA1*  / SXT | *qnrS1; gyrA S83L, parC S80R*  / NAL, CIP, LVX | *mcr-1.1;*  *pmrA S39I*  / CST | - / - |
| LREC-170 | -  / TIC, AMP | - / - | *mdf(A)* | -  / MIN*, DOX | - / - | - / - | - / - | *mcr-1.1*  */* CST | - / - |
| LREC-171 | *bla_TEM-1B_*  / TIC, AMP, SAM, AMC* | *aadA1, aph(3')-Ia, aph(3'')-Ib, aph(6)-Id*  */ -* | *mdf(A)* | *tet(B)*  / MIN, DOX | - / - | *sul1*  / - | *gyrA S83L, gyrA D87Y, parC S80R*  / NAL, CIP, LVX | *mcr-1.1*  */* CST | - / - |
| LREC-172 | *bla_CTX-M-14_*  / TIC, AMP, SAM, CFZ,CXM, CTX, FEP, ATM | *aadA1*  / GEN*, TOB* | *mdf(A)* | *tet(A)*  / DOX* | *catA1*  / CHL | *sul1; dfrA1*  / SXT | *gyrA S83L*  / NAL, CIP, LVX | *mcr-1.1*  */* CST | - / - |
| LREC-174 | *bla_TEM-1B_*  / TIC, AMP, SAM | *aadA1, aadA2*  / GEN, TOB | *mdf(A)* | - / - | *catA1, cmlA1*  / CHL | *sul3*  / - | *gyrA S83L*  / NAL | *mcr-1.1*  */* CST | - / - |
| LREC-175 | - / - | - / - | *mdf(A)* | - / - | - / - | - / - | *gyrA S83L*  / NAL | *mcr-1.1*  */* CST | NIT* |
| LREC-178 | *bla_TEM-1B_*  **/** TIM, AMP | *aadA1, aadA2*  / GEN, TOB* | *mdf(A), mph(B)* | - / - | *cmlA1*  / CHL | *sul1, sul3; dfrA1*  / SXT | *gyrA D87G*  / - | *mcr-1.1;*  *pmrB V161G*  / CST | - / - |
| LREC-151 | - / - | *aph(3')-Ia*  **/ -** | *mdf(A)* | - / - | - / - | - / - | *gyrA S83L*  / NAL* | *mcr-1.10*  / CST | - / - |
| LREC-136 | *bla_TEM-1B_*  / TIC, AMP, SAM, AMC | *aadA1, aadA2; aph(3')-Ia*  / - | *mdf(A)* | -  / DOX* | *cmlA1*  / CHL | *sul3*  / - | *gyrA S83L*  / NAL | *mcr-4.1*  / CST | - / - |
| LREC-131 | *bla*_SHV-12_  / TIC, AMP, CFZ, CXM, CTX, CAZ, ATM | *aadA2, aph(3’)-Ia*  / - | *mdf(A)* | *tet(B)*  / MIN*, DOX | *cmlA1*  / CHL | *sul3; dfrA12*  / SXT | *gyrA S83L, gyrA D87G, parC S80I*  / NAL, CIP, LVX | *mcr-4.2*  / CST | - / - |
| LREC-132 | *bla_TEM-1B_*  / TIC, AMP, AMC | *aac(3)-IIa, aac(3)-IVa, aadA1, aph(3’)-Ia, aph(3'')-Ib, aph(4)-Ia*  / GEN, TOB | *mdf(A), mph(B)* | *tet(B)*  / MIN, DOX | *catA1*  / CHL | *sul1; dfrA1*  / SXT | *gyrA S83L, gyrA D87G, parC S80I*  / NAL, CIP, LVX | *mcr-4.2;*  *pmrB V161G*  / CST | - / - |
| LREC-133 | - / - | - / - | *mdf(A)* | -  / MIN, DOX | - / - | -  / SXT | *gyrA S83L, parC S80R*  / NAL, CIP | *mcr-4.2*  / CST | - / - |
| LREC-134 | - / - | *aac(3)-IVa, aph(3'')-Ib, aph(4)-Ia, aph(6)-Id*  / GEN, TOB | *mdf(A)* | - / - | - / - | - / - | *gyrA* D87N  / - | *mcr-4.2*  / CST | - / - |
| LREC-137 | *bla_TEM-1B_*  / TIC, AMP | *aadA1*  / GEN, TOB | *mdf(A)* | *tet(B)*  / MIN, DOX | -  / CHL* | *sul3; dfrA1*  / SXT | *gyrA S83L*  / NAL, CIP* | *mcr-4.2;*  *pmrB V161G*  / CST | - / - |
| LREC-138 | - / - | *aac(3)-IV, aph(3'')-Ib, aph(4)-Ia, aph(6)-Id*  / - | *mdf(A)* | *tet(B)*  / MIN*, DOX | *floR*  / CHL | - / - | *gyrA S83L, gyrA D87G, parC S80I*  / NAL, CIP, LVX | *mcr-4.2;*  *pmrB V161G*  / CST | - / - |
| LREC-139 | - / - | *aadA2*  / - | *mdf(A), inu(F)* | - / - | - / - | - / - | *gyrA S83L, gyrA D87N, parC S80I*  / NAL, CIP, LVX | *mcr-4.2*  / CST | - / - |
| LREC-140 | *bla_CTX-M-32_, bla_TEM-1B_*  / TIC, AMP, CFZ, CXM, CTX, CAZ, FEP, ATM | *aac(3)-IV, aadA1, aph(3'')-Ib, aph(4)-Ia*  / GEN*, TOB* | *erm(B), mdf(A), mph(B)* | *tet(B), tet(M)*  / MIN, DOX | *catA1*  / CHL | *sul1; dfrA1*  / SXT | *gyrA S83L, gyrA D87G, parC S80I*  / NAL, CIP, LVX | *mcr-4.2;*  *pmrB V161G*  / CST | - / - |
| LREC-142 | *bla_TEM-1B_*  / TIC, AMP | *aadA1, aadA2, acc (3)-IV, aph(3’)-Ia, aph(3'')-Ib, aph(4)-Ia, aph(6)-Id*  / GEN*, TOB | *mdf(A), inu(G)* | *tet(B)*  / DOX | *cmlA1, catA1*  / CHL | *sul1, sul3; dfrA1*  / SXT | *gyrA S83L, gyrA D87G, parC S80I*  / NAL, CIP, LVX | *mcr-4.2;*  *pmrB V161G*  / CST | NIT* |
| LREC-143 | - / - | *aadA1, aph(3’)-Ia*  / - | *mdf(A)* | *tet(B)*  / MIN, DOX | *catA1*  / CHL | *sul1; dfrA1*  / SXT | *gyrA S83L, parC S80R*  / NAL, CIP | *mcr-4.2*  / CST | - / - |
| LREC-156 | *bla_CTX-M-14_ bla_TEM-1B_*  / TIC, AMP, CFZ, CXM, CTX, FEP | *aac(3)-IV, aadA1, aadA2, aph(3’)-Ia, aph(3'')-Ib, aph(4)-Ia, aph(6)-Id*  / GEN*, TOB | *mdf(A)* | *tet(B)*  / MIN, DOX | *cmlA1, catA1*  / CHL | *sul1, sul3; dfrA1*  / SXT | *gyrA S83L, gyrA D87G, parC S80I*  / NAL, CIP, LVX | *mcr-4.2;*  *pmrB V161G*  / CST | - / - |
| LREC-135 | *bla_TEM-1A_*  / TIC, AMP | *aac(3)-IVa, aph(3'')-Ib, aph(4)-Ia, aph(6)-Id*  / GEN*, TOB | *mdf(A)* | - / - | - / - | - / - | *gyrA S83L, gyrA D87G, parC S80I, parE L416F*  / NAL, CIP, LVX | *mcr-4.5;*  *pmrB V161G*  / CST | - / - |
| LREC-146 | *bla_OXA-1_, bla_TEM-1A_*  / TIC, AMP, SAM, AMC | *aac(3)-IV, aadA1, aph(3'')-Ib, aph(4)-Ia, aph(6)-Id*  / GEN, TOB | *mdf(A)* | - / - | *floR*  / CHL | *sul1, sul2*  / SXT | *gyrA S83L, gyrA D87G, parC S80I*  / NAL, CIP, LVX | *mcr-4.5;*  *pmrB V161G*  / CST | - / - |
| LREC-152 | *bla_TEM-1A_*  / TIC, AMP, SAM | *aac(3)-IV, aph(3’)-Ia, aph(3'')-Ib, aph(4)-Ia, aph(6)-Id*  / GEN, TOB | *mdf(A)* | - / - | - / - | - / - | *gyrA S83L*  / NAL | *mcr-5.1;*  *pmrB V161G*  / CST | - / - |
| LREC-177 | - / - | *aadA1, aph(3’)-Ia*  / - | *mdf(A)* | *tet(B)*  / MIN, DOX | - / - | *dfrA1*  / SXT | *gyrA S83L*  / NAL | *mcr-5.1;*  *pmrB V161G*  / CST | - / - |
| LREC-141 | *bla_TEM-1A_*  / TIC, AMP, SAM, AMC* | *aac(3)-IIa, aadA1,aph(3')-Ia, aph(3'')-Ib, aph(6)-Id*  / GEN, TOB | *mdf(A)* | *tet(A)*  / DOX* | - / - | *sul1, sul2, sul3; dfrA1*  / SXT | *gyrA S83L, gyrA D87G, parC S80I, parE L416F*  / NAL, CIP, LVX | *mcr-1.1, mcr-4.2; pmrB V161G*  / CST | - / - |
| LREC-163 | *bla_TEM-1A_*  / TIC, AMP, SAM, AMC* | *aadA1, aadA17, aph(3'')-Ib, aph(6)-Id*  / GEN, TOB* | *inu(F), mdf(A)* | *tet(A)*  / - | - / - | *sul1, sul3; dfrA1*  / SXT | *gyrA S83L, gyrA D87G, parC S80I*  / NAL, CIP, LVX | *mcr-1.1, mcr-4.2; pmrB V161G*  / CST | - / - |

^1^AMC: amoxicillin/clavulanate, AMP: ampicillin, AMP/SAM: ampicillin-sulbactam, ATM: aztreonam, CAZ: ceftazidime, CHL: chloramphenicol, CIP: ciprofloxacin, CST: colistin, CTX: cefotaxime, CXM: cefuroxime, CFZ: cefazolin, DOX: doxycycline, FEP: cefepime, FOF: fosfomycin, GEN: gentamicin, LVX: levofloxacin, MI: minocycline, NAL: nalidixic acid, NIT: nitrofurantoin, SXT: trimethoprim/sulfamethoxazole, TIC: ticarcillin, TOB: tobramycin. Antimicrobial susceptibility was interpreted according to the CLSI (CLSI, 2019). The intermediate resistance is indicated with asterisk (*). ^2^Resistance to macrolides was not included in the phenotypic antimicrobial susceptibility tests.
